# Clinical Utility of Genetic Testing with Geographical Locations in ADPKD: Describing New Variants

**DOI:** 10.3390/jcm13061751

**Published:** 2024-03-18

**Authors:** Carmen García Rabaneda, María Luz Bellido Díaz, Ana Isabel Morales García, Antonio Miguel Poyatos Andújar, Juan Bravo Soto, Anita Dayaldasani Khialani, Margarita Martínez Atienza, Rafael Jose Esteban de la Rosa

**Affiliations:** 1 Servicio de Análisis Clínicos, Valle de los Pedroches Hospital, 14400 Pozoblanco, Spain; 2 Servicio de Análisis Clínicos Granada, Hospital Universitario Virgen de las Nieves, 18014 Granada, Spain; marialuzbellidodiaz@gmail.com (M.L.B.D.); antoniom.poyatos.sspa@juntadeandalucia.es (A.M.P.A.); margarita.martinez.atienza.sspa@juntadeandalucia.es (M.M.A.); 3 Nefrología Granada, Hospital Universitario San Cecilio, 18016 Granada, Spain; amoralesg@senefro.org; 4 Nefrología Granada, Hospital Universitario Virgen de las Nieves, 18014 Granada, Spain; jabravosoto@gmail.com(J.B.S.); rafaelj.esteban@gmail.com (R.J.E.d.l.R.); 5 Análisis Clínicos Málaga, Hospital Regional Universitario Carlos Haya, 29010 Málaga, Spain; anniedayal@gmail.com; 6 Biosanitary Research Institute of Granada (ibs.GRANADA), 18012 Granada, Spain

**Keywords:** ADPKD, geographical location, new variants

## Abstract

**Background:** Our study aims to comment on all ADPKD variants identified in our health area and explain how they are distributed geographically, to identify new variants, and relate the more frequent variants with their renal phenotype in terms of kidney survival. **Materials and Methods:** We identified patients suffering from ADPKD in a specialized consultation unit; genealogical trees were constructed from the proband. According to the ultrasound-defined modified Ravine–Pei criteria, relatives classified as at risk were offered participation in the genetic study. Socio-demographic, clinical, and genetic factors related to the impact of the variant on the survival of the kidney and the patient, such as age at RRT beginning and age of death, were recorded. **Results:** In 37 families, 33 new variants of the *PKD1* gene were identified, which probably produce a truncated protein. These variants included 2 large deletions, 19 frameshifts, and 12 stop-codons, all of which had not been previously described in the databases. In 10 families, six new probably pathogenic variants in the *PKD2* gene were identified. These included three substitutions; two deletions, one of which was intronic and not associated with any family; and one duplication. A total of 11 missense variants in the *PKD1* gene were grouped in 14 families and classified as probably pathogenic. We found that 33 VUS were grouped into 18 families and were not described in the databases, while another 15 were without grouping, and there was only 1 in the *PKD2* gene. Some of these variants were present in patients with a different pathogenic variant (described or not), and the variant was probably benign. Renal survival curves were compared to nonsense versus missense variants on the *PKD1* gene to check if there were any differences. A group of 328 patients with a nonsense variant was compared with a group of 264 with a missense variant; mean renal survival for truncated variants was lower (53.1 ± 0.46 years versus non-truncated variant 59.1 ± 1.36 years; Log Rank, Breslow, and Tarone Ware, *p* < 0.05). **Conclusions:** To learn more about ADPKD, it is necessary to understand genetics. By describing new genetic variants, we are approaching creation of an accurate genetic map of the disease in our country, which could have prognostic and therapeutic implications in the future.

## 1. Introduction

Autosomal dominant polycystic kidney disease (ADPKD), classified with the international codes 753.12, 753.13 (ICD-9) and Q61.2, Q61.3 (ICD-10), is the most common hereditary nephropathy, causing renal failure and requiring renal replacement therapy (RRT). It is estimated to affect at least 10 million people worldwide and accounts for up to 10% of all patients on dialysis and/or undergoing transplantation [1].

Early diagnosis of ADPKD is mainly established by ultrasonography (US). However, in some situations, such as younger individuals and those with *PKD2* mutations, US may be insufficient to provide a definitive diagnosis [2,3]. In such situations, genetic analysis has become crucial in confirming the presence of ADPKD, leading to an expansion of databases containing pathogenic variants associated with this condition. This has helped address the issue of underdiagnosis in the past.

The age at which individuals with ADPKD progress to end-stage renal disease (ESRD) and the severity of the disease can vary significantly, even among members of the same family. This variability is influenced by the specific variants affecting the genes responsible for encoding polycystin 1 and 2 proteins (PC1 and PC2) [4]. Patients with *PKD1* variants reach renal failure earlier than those with *PKD2* ones [5].

The purpose of our study is to analyze and describe all the ADPKD variants identified in our health area. Additionally, we aim to explore the geographic distribution of these variants, identify any new variants, and examine the relationship between the most common variants and the renal phenotype in terms of kidney survival.

## 2. Materials and Methods

### 2.1. Patients

A total of 1187 ADPKD patients were included in the study. Among them, 1096 patients were from 295 unrelated families, and genograms were performed to identify all affected members and their places of residence. Additionally, 90 individuals were not grouped into families, and genograms were not performed for them. The diagnosis of ADPKD was established based on ultrasound criteria [2]. Patients were selected in ADPKD monographic consultations in Virgen de las Nieves and San Cecilio Hospitals in Granada during the period 2010–2019. All patients invited to participate in the study were provided with detailed information about the study’s purpose and signed informed consent forms.

Socio-demographic, clinical, and genetic factors related to the impact of the variant on the survival of the kidney and the patient were recorded. This included information such as age at RRT beginning and age of death.

### 2.2. Blood Collection and Genetic Analysis

An amount of 3–5 mL of peripheral blood was obtained from all the participants and stored in a container with EDTA anticoagulant, in the extraction room of our hospital.

The genetic studies were carried out, firstly, in an external laboratory using the NextGeneDx^®^ massive sequencing (NGS) panel from Illumina San Diego, CA, USA, which includes the *PKD1*, *PKD2*, and *GANAB* genes, using the following methodology:

1. Extraction of genomic DNA from sample blood cells;
2. Preparation of libraries, using the Nextera XT kit (Illumina San Diego, CA, USA.);
3. Sequencing of the libraries (2 × 150), with the MiSeq sequencer (Illumina);
4. Bioinformatic analysis of the sequences obtained, using the Sophia program.

All the variants found were confirmed by Sanger sequencing.

Starting in 2018, our hospital began to implement massive sequencing (NGS), and a panel for hereditary kidney disease was launched, which now includes 44 genes, including *PKD1*, *PKD2,* and *GANAB*, using the following methodology:

Genomic DNA extraction: It was extracted from blood cells in the Qiagen kit.

Building the library: The Sophia Genetics Nephropathies Solution Kit was used, following the manufacturer’s instructions.

Sequencing: An Illumina MiSeq sequencer was used. Captured sequences were amplified, and FASTQ, BAM, and VCF files generated. The minimum base reading and amplicon coverage were 50× and 100×, respectively.

Annotation of variants: These were interpreted in the SOPHIA DDM platform and designed for the analysis and protection of clinical NGS data in routine diagnosis.

To evaluate the pathogenicity of the variants found, population databases such as the Human Gene Mutation Database (HGMD), Clinvar, GenomeAD, LOVD, and the main ADPKD variant database were used.

### 2.3. Statistical Analysis 

Geno-Pro software 2020 3.1.0.1 was used to build the ADPKD family trees; we assume that the variant that is identified in a family is the same in the rest of the affected members. Data are expressed in terms of mean ± SD, range, median, and %, as appropriate. Renal and patient survival studies were performed using a Kaplan–Meier test, and a Mantel–Cox test was used to compare survival curves. Significance was considered when *p* < 0.05. The number of patients selected was the minimum to ensure statistical power. Statistical analysis was performed using the SPSS 15.0 package.

## 3. Results

### 3.1. Molecular Analysis and Geographical Location of the Family

Table 1 shows new nonsense variants, which were identified and considered as pathogenic in the *PKD1* and *PKD2* genes, according to the criteria of the American College of Medical Genetics (ACMG), together with their geographical locations. In 37 families, 33 new variants on *PKD1* gene were identified, which probably produce a truncated protein. These variants included 2 large deletions, 19 frameshifts, and 12 stop-codons, all of which had not been previously described in the databases. The c.11294_11313del20 (p. Pro3765Argfs*44) variant was the most prevalent, present in four families located in Granada and Barcelona.

In 10 families, six new probably pathogenic variants in the *PKD2* gene were identified. These included three substitutions; two deletions, one of which was intronic and not associated with any family; and one duplication. c.295G>T (p. Glu99*) was the most frequent variant present in four unrelated families from Motril, and the c.1807dupA variant (p. Met603Asnfs*23) was present in two unrelated families. All of these variants give rise to a non-functional truncated protein.

Table 2 shows 11 missense variants in the *PKD1* gene that were grouped in 14 families, which cause one amino acid to change to another thus producing a change in the structure of the protein, and classified as probably pathogenic following the ACMG criteria. The variant c.7292T>A has been reported as a founder effect by our group (6). The family trees are shown in the Supplementary Materials (Supplementary Figures S1–S4).

We did not locate any undescribed missense variants in the *PKD2* gene. 

Table 3 shows the VUS not described in the databases, 33 of which were grouped into 18 families, while another 15 were without grouping. These had not been described in databases but were classified as VUS by in silico predictors, and there was only one in the *PKD2* gene. Some of these variants were present in patients with a different pathogenic variant (described or not), and the variant is probably benign. Examples include Fam 58 with pathogenic variant c.11252A>C(p.Gln3751Pro) and VUS c.127C>G(p.Pro43Ala) or Fam 53 with probably pathogenic variant c.9364A>T(pIle3122Phe) and VUS c.6913C>G(p.Gln2305Glu). The second variant identified is probably benign, although in silico predictors defined it as a VUS. 

All variants with their geographical locations can be found in the Supplementary Materials (Supplementary Figure S5).

### 3.2. Renal Survival Analysis 

Table 4 shows the data collected from families with more than 5 PKD individuals. In order to enhance statistical power, we included all the variants considered pathogenic or probably pathogenic, described or not, which were identified in the family members. The mean age of initiation of RRT shows a wide range, between 48 and 78 years. Table 5 shows the renal survival analysis in patients with variants in *PKD2*. Due to the scarce data obtained and the few variants with a high number of patients for statistical analysis with sufficient statistical power, we cannot compare truncating and non-truncating variants for this gene.

**Figure 1:**
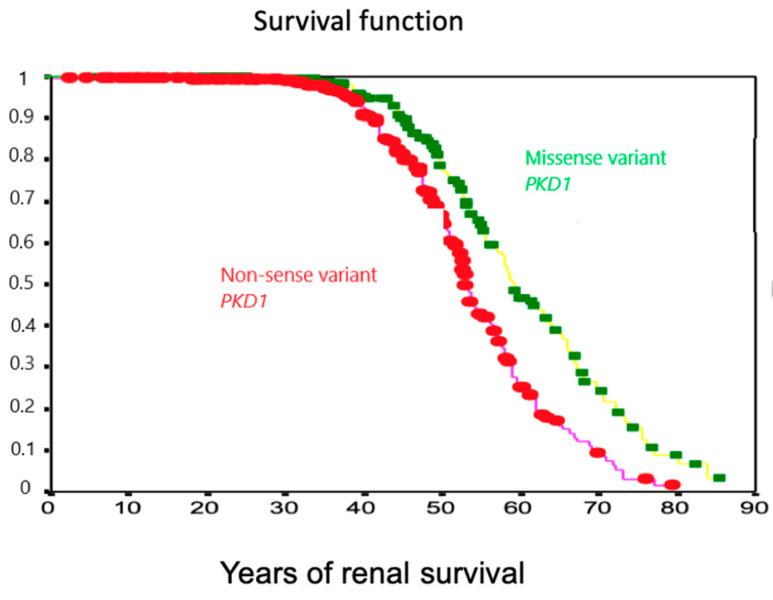
Renal survival in nonsense versus missense variants in the *PKD1* gene.

Renal survival curves were compared using nonsense versus missense variants on the *PKD1* gene to check if there were any differences. A group of 328 patients with a nonsense variant was compared with a group of 264 with a missense variant, and the result is shown in Figure 1; mean renal survival for truncated variants was lower (53.1 ± 0.46 years versus non-truncated variant 59.1 ± 1.36 years; Log Rank, Breslow, and Tarone Ware, *p* < 0.05).

Table 6 summarizes the age at which RRT was started, as well as the age at which death occurred in the patients according to type of variant on *PKD1* gene: in missense variants, renal survival in terms of RRT was upper (*p* < 0.05), as was age of death (*p* < 0.05).

## 4. Discussion

The value of our work lies not only in the fact that we have identified a large number of new nonsense, missense, and VUS variants in *PKD1* and *PKD2* genes, but also that we offer valuable information on geographical location. It is of the utmost importance that all the working groups researching this disease share, publish, and register the variants found in patients, especially VUS, as this allows us to submit them to monitoring, reclassification of variants, and research. This will help us to characterize them with all the contributions of the different study groups and perform segregation studies. The usefulness of identifying other members of the families in other locations will allow us to include families that were unaware of their genetic profile, because there are individuals from the same families that are not identified in the genogram, as well as to locate a founder effect in isolated areas.

In addition, in small families, knowledge of genetic studies in other families with the same variant will allow us to make comparisons and, in the case of VUS, will increase the power of segregation studies or a founder effect in isolated areas. In our study, some of the variants we found were classified as VUS in the databases, but as pathogenic or probably pathogenic in families with another variant, such as Fam 53 or 58 (see Table 3). Segregation studies are necessary for those couples who present VUS but have family histories of ADPKD, and who wish to access preimplantation genetic testing (PGT) to have healthy children. If there are more families with this variant and the presence of the disease is demonstrated in all of them, access to these therapies will be easier.

The new ADPKD variants identified are mainly located in the province of Granada, and as we performed the genograms, we were able to collect information on other members of these families living in other geographical locations who were affected. This information has allowed us to identify ‘hot spots’ such as the presence of the c.295G>T variant in the *PKD2* gene in four unrelated ADPKD families in Motril, a population of 60,592 inhabitants, which could represent a founder effect [6].

Finally, in some variants, those with more than five members’ limbs affected, we offer information on renal and patient survival. The follow-up of the patients over the years has allowed us to obtain data on the loss of renal function and age of death. With the statistical power provided by a large number of families studied, the aim was to provide a global view of the different effects that occur at the renal level, depending on the type of family variant and even within the same family. These will have important repercussions on access to treatment for affected patients. The average age of initiation of RRT for *PKD1* variants ranges between 48 and 78 years, and around 72 years for *PKD2* variants. 

After carrying out family genetic studies and grouping the families in family trees, we have characterized numerous variants, many of which had not been previously identified, since they have not been registered in databases [7]. The latest prevalence studies have shown that a discrepancy exists between the estimated genetic prevalence and the prevalence of ADPKD. This suggests that patients with a mild form of the disease or young individuals are often not diagnosed, either because imaging tests are not often performed, or because the results may be not noteworthy according to Ravine and Pei’s criteria [3]. In Olmsted County, a study was performed to investigate this discrepancy, in which radiology reports were systematically reviewed to identify patients with possible ADPKD. After the review, the probable cases were analyzed, and the incidence and point prevalence increased. However, these were lower than the genetic prevalence estimated in population sequencing databases. This indicates that there are patients with possible ADPKD who may have mild variants in *PKD1* or *PKD2* or variants in other ADPKD genes such as GANAB or DNAJB11, because only 18% of patients have probably pathogenic nonsense variants in *PKD1*, according to Lanktree et al. [8]. Our study shows that when families are studied using genetic studies and family trees, the incidence in our health area is higher than the incidence published in other studies, and the number of underdiagnosed patients is lower. Our data, collected from family trees, have allowed us to identify families not only in our health area, but also in other parts of the country, with many variants found in Granada and Barcelona, but also in Jaen, Córdoba, and Seville [7].

In addition, the incorporation of genetic studies into clinical practice will improve the diagnostic yield; in our case, out of the 295 families with ultrasound suspicion identified in the genetic study, 225 were in *PKD1* and 19 in *PKD2* genes, with a diagnostic yield of 82.7%. Of these, 78 variants in *PKD1* have not previously been described in databases, and in *PKD2*, 24 patients were found with 7 variants not described in databases. Currently, we are contributing our data to the Clinvar database to be included in future updates. This will allow geneticists and nephrologists to characterize a larger number of families, and the incorporation of these variants will also allow us to build a genetic map of ADPKD in our health area.

Genotype–phenotype studies have shown that the gene and type of a variant are key factors to explain many of the clinical complications in ADPKD patients [5,9]. In 2019, Cornec-Le Gall et al. noted that patients with *PKD2* variants have a more favorable renal prognosis than patients with *PKD1* variants, with a median age at ESRD around 79 years with variant *PKD2*, versus 58 years with variant *PKD1* [10,11]. The GENKYST study highlights the fact that patients with *PKD1* variants can be divided into two groups: those with a nonsense variant (*PKD1N*, associated with a worse renal prognosis) and those with a missense variant (*PKD1M*). The median ages for ESRD for patients with these two variants were 55.6 years and 67.9 years, respectively [1,10]. The data obtained by our group, reflected in Table 6, are similar for the *PKD1M* variants compared to that review, although the age is significantly lower for those with *PKD1N*: 51 years.

Most studies reflect the fact that ADPKD patients develop ESRD around 70 years old (2). However, our data show that some patients access RRT earlier, which demonstrates the variability of kidney disease in ADPKD (2). Our results suggest that genetic modifiers that affect the onset of this renal disease may be present in some families, such as the co-inheritance of a weak allele of *PKD1* or *PKD2* or a variant in a cis position concerning the major variant [1,8,10,12,13]. We must also take into account those patients with large deletions that can affect nearby genes, such as the tuberous sclerosis complex genes, TSC2 adjacent to *PKD1*, since they can affect both genes, causing a severe phenotype [14]. Concomitant nephropathies have also been identified in patients who developed ESRD earlier than expected based on genotypes [14,15].

The evaluation of the progression risk in ADPKD has acquired clinical importance since the approval of Tolvaptan, the first pharmacological treatment authorized exclusively for patients with high progression risk based on a prognostic algorithm, the Predicting Renal Outcomes in Polycystic Kidney Disease (PROPKD Score), which combines predictive genetic factors (*PKD1N* vs. *PKD1M/PKD2*) with clinical information (gender, age at diagnosis of hypertension, and first urological diagnosis). It provides a stratification of the risks of decreased estimated glomerular filtration rate and progression to renal failure [10,11,13]. However, data from our study show that some of the supposedly less aggressive variants cause mild cases of the disease, and yet they also produce early cases of RRT, as in the case of the variant c.8819C>T (p.Pro2940Leu), where the age at which the loss of kidney function occurs is 63.66 (59.54–67.69), compared to the case of the pathogenic variant described c.9202-16G>A (p.Pro3067fs*), where the age at which renal loss occurs is 71.09 (61.77–80.40). 

For the *PKD2* gene, we obtained data from only two families, because the presence of variants in this gene is less significant. Those acquired were nonsense variants (c.1445delT p.Phe482Serfs*32, c.1807dup p.Met603Asnfs*23), and the ages of access to RRT were 73.60 (63.43–83.77) and 72.87 (66.09–79.66), respectively. The median age for those patients is near to the value for variants in *PKD1*. 

These data indicate that the assessment of rapid progress according to the type of variant should be reviewed, as certain patients are being excluded and deprived of the benefits of this new therapy. Genetic variability and other factors can influence the functional capacity of the protein and the progression of the disease, and, although intrafamilial renal disease discordance among affected relatives is a recognized feature in ADPKD patients, defining its progression is challenging. Therefore, this should prompt nephrologists and geneticists to search for additional genetic and environmental factors that could help explain their choice of patients for therapy.

In summary, our work to identify a large number of new variants associated with ADPKD has provided us with additional information about their corresponding geographical location, which will help to build a genetic map of the disease. Constructing this ADPKD genetic map will help us to understand the disease and its prognosis better, identify disease hot spots, offer better access to current and future specific therapies, and facilitate the use of PGT in couples who wish to have healthy children. 

## Figures and Tables

**Table 1:** New, probably pathogenic nonsense variants in *PKD1* and *PKD2* genes and geographical location of the family. Exon (E), Intron (I).

Family Number	Gene	c.DNA Designation	Protein Change	Exon/Intron	Number Patients Affected	Geographical Location
Fam1	*PKD1*	c.160_166dup	(p.Leu56Profs*60)	E1	1	GRANADA
Fam11	*PKD1*	c.419delC	(p.Ala140Glyfs*150)	E4	1	GRANADA
Fam20	*PKD1*	c.1548G>A	(p.Trp516*)	E7	2	GRANADA
Fam12	*PKD1*	c.2591delG	(p.Gly864Alafs*34)	E11	7	GRANADA-SEVILLA
Fam25 Fam26	*PKD1*	c.2702G>A	(p.Trp901*)	E11	7	GRANADA-GRAN CANARIA-MONTEVIDEO
Fam21	*PKD1*	c.2847A>T	(p.Lys949*)	E11	1	GRANADA-CIUDAD REAL-MADRID
Fam32	*PKD1*	c.3067C>T	(p.Gln1203*)	E13	3	GRANADA
Fam56	*PKD1*	c.3032dupT	(p.Thr1012fs*89)	13	3	GRANADA
Fam33	*PKD1*	c.3162-?_6915+?del	p.-	I 14-15	9	GRANADA
Fam27 Fam28	*PKD1*	c.3346C>T	(p.Gln1116*)	E15	2	GRANADA-MALAGA-VALENCIA-MURCIA
Fam29	*PKD1*	c.5007G>A	(p.Trp1669*)	E15	1	JAEN
Fam13	*PKD1*	c.5013_5031del	(p.Asp1671Glufs*45)	E15	16	JAEN-MALLORCA-SEVILLE
Fam22	*PKD1*	c.5637C>G	(p.Tyr1879*)	E15	1	GRANADA
Fam2	*PKD1*	c.5912dupT	(p.Ser1972Glufs*18)	E15	2	GRANADA-BADAJOZ
Fam14	*PKD1*	c.6473delA	(p.Gln2158Argfs*3)	E15	1	GRANADA
Fam30	*PKD1*	c.6281G>A	(p.Trp2094*)	E15	1	GRANADA
Fam23	*PKD1*	c.7480G>T	(p.Glu2494*)	E18	7	GRANADA-SEVILLE
Fam3	*PKD1*	c.7733_7734dupAC	(p.Gly2579Thrfs*42)	E20	4	GRANADA
Fam4	*PKD1*	c.7784_7785dup	(p.Leu2596Cysfs*25)	E20	8	GRANADA-JAEN-CADIZ
Fam5	*PKD1*	c.8326_8330dup	(p.Gly2778Trpfs*99)	E23	1	HUELVA
Fam6	*PKD1*	c.8327_8334dup	(p.Glu2779Trpfs*9)	E23	2	GRANADA-CORDOBA-JAEN
Fam15	*PKD1*	c.8579delA	(p.Gln2860Argfs*15)	E23	8	GRANADA-BARCELONA
Fam34	*PKD1*	Deletion 117 aac (MLPA)	p.-	E31	4	GRANADA
Fam31	*PKD1*	c.10306C>T	(p.Gln3436*)	E33	7	GRANADA-CADIZ-BARCELONA
Fam16 Fam17	*PKD1*	c.10441delG	(p.Val3481Serfs*46)	E34	12	GRANADA
Fam7	*PKD1*	c.10759dupG	(p.Ala3587Glyfs*40)	E36	3	GRANADA-MALAGA
Fam24	*PKD1*	c.11266G>T	(p.Glu3756*)	E39	2	GRANADA
Fam18 Fam19	*PKD1*	c.11294-11313del20	(p.Pro3765Argfs*44)	E40	35	GRANADA-BARCELONA
Fam8	*PKD1*	c.11378_11379dup	(p.Thr3794Glyfs*33)	E40	1	GRANADA-SENEGAL
Fam9	*PKD1*	c.12056dupT	(p.Leu4019Phefs*138)	E44	1	GRANADA-AVILA-BARCELONA-MADRID
Fam10	*PKD1*	c.12246_12252dup	(p.Leu4085Valfs*74)	E45	3	BARCELONA-GRANADA-CORDOBA
Fam92	*PKD1*	c.12431 _12439delGCAAGGTCA	(p.Ser4144fs*50)	E45	2	GRANADA-LONDON
Fam35	*PKD2*	c.242C>A	(p.Ser81*)	E1	1	GRANADA-ROMANIA
Fam36 Fam37 Fam 96 Fam 97	*PKD2*	c.295G>T	(p.Glu99*)	E1	12	GRANADA (Motril)
Fam 39	*PKD2*	c.411delG	(p.Ser138Alafs*95)	E1	4	GRANADA
Fam96	*PKD2*	c.584+1delG	-	I 4		
Fam40 Fam41 Fam42 Fam43	*PKD2*	c.1807dupA	(p.Met603Asnfs*23)	E8	13	GRANADA-JAEN-MALAGA-MALLORCA
Fam38	*PKD2*	c.1864C>T	(p.Gln622*)	E8	4	JAEN

**Table 2:** New, probably pathogenic missense variants in *PKD1* gene and geographical location of the family.

Family Number	Gene	c.DNA Designation	Protein Change	Exon/Intron	Patients Affected	Geographical Location
Fam44	*PKD1*	c.1147T>C	(p.Ser383Pro)	E5	1	GRANADA-VALLADOLID
Fam45	*PKD1*	c.1261C>T	(p.Arg421Cys)	E6	2	GRANADA
Fam46	*PKD1*	c.3719A>G	(p.Asn1240Ser)	E15	2	GRANADA
Fam47 Fam48 Fam49 Fam50	*PKD1*	c.7292T>A	(p.Leu2431Gln)	E18	66	GRANADA-CORDOBA-BARCELONA
Fam51	*PKD1*	c.7553G>T	(p.Arg2518Leu)	E19	1	GRANADA
Fam53	*PKD1*	c.9364A>T	(p.Ile3122Phe)	E26	2	GRANADA
Fam54	*PKD1*	c.9380G>T	(p.Gly3127Val)	E26	8	GRANADA-VALENCIA
Fam55	*PKD1*	c.11639T>G	(p.Leu3880Arg)	E42	1	GRANADA
Fam52	*PKD1*	c.11953T>C	(p.Ser3985Pro)	E43	5	GRANADA
Fam57	*PKD1*	c.11961_11966delinsAGA	(p.Arg3988_Gly3989delinsAsp)	E43	1	GRANADA

**Table 3:** Variants of unknown significance (VUS), not described, in *PKD1* or *PKD2* genes and geographical location of the family.

Family Number	Gene	c.DNA Designation	Protein Change	Exon/Intron	Number Patients Affected	Geographical Location
Fam58	*PKD1*	c.127C>G	(p.Pro43Ala)	E1	1	GRANADA
Fam60	*PKD1*	c.1548G>T	(p.Trp516Cys)	E7	1	GRANADA
Fam92	*PKD1*	c.1634T>A	(p.Leu545His)	E8	2	GRANADA-LONDON
Fam62	*PKD1*	c.1779A>T	(p.Glu593Asp)	E9	1	GRANADA-BARCELONA-IBIZA
Fam63	*PKD1*	c.1799G>A	(p.Arg611Trp)	E9	1	JAÉN-GRANADA
Fam64	*PKD1*	c.2563G>C	(p.Ala855Pro)	E11	1	JAÉN
Fam65	*PKD1*	c.3425G>C	(p.Arg1142Pro)	E15	3	GRANADA
Fam66	*PKD1*	c.3490G>A	(p.Gly1164Arg)	E15	2	GRANADA
Fam69	*PKD1*	c.4988_4990del	(p.Ser1663del)	E15	10	GRANADA
Fam71	*PKD1*	c.5125G>T	(p.Asp1709Tyr)	E15	1	GRANADA
Fam72	*PKD1*	c.5950A>G	(p.Ile1984Val)	E15	1	GRANADA
Fam73	*PKD1*	c.6005T>A	p.Val2002Asp)	E15	11	GRANADA-TOLEDO-MADRID
Fam 53	*PKD1*	c.6913C>G	(p.Gln2305Glu)	E15	1	GRANADA
Fam18 Fam 19	*PKD1*	c.7210-7C>T	(p.-)	I 17	1	GRANADA-BARCELONA
Fam76	*PKD1*	c.7236_7238delCAA	(p.Asn2412del)	E18	4	GRANADA
Fam77	*PKD1*	c.7261A>C	(p.Thr2421Pro)	E18	1	GRANADA-CORDOBA
Fam 55	*PKD1*	c.8191G>A	(p.Val2731Met)	E23	1	GRANADA
Fam52	*PKD1*	c.9083A>C	(p.Glu3028Ala)	E25	5	GRANADA
Fam82	*PKD1*	c.9575G>T	(p.Ser3192Ile)	E28	8	GRANADA-CORDOBA
Fam84	*PKD1*	c.9782A>G	(p.His3261Arg)	E29	1	CADIZ-GRANADA
Fam85	*PKD1*	c.10873G>A	(p.Asp3625Asn)	E37	1	CORDOBA-GRANADA-MADRID-VALLADOLID
Fam86	*PKD1*	c.11518_11529del	(p.His3840_Leu3843del)	E41	1	GRANADA
Fam88	*PKD1*	c.11545G>C	(p.Ala3849Pro)	E42	1	GRANADA
Fam93	*PKD1*	C.12320G>A	(p.Arg4107His)	E45	1	GRANADA-SALAMANCA-VALLADOLID-MALLORCA
Fam95	*PKD2*	c.2522+8T>G	(p.-)	I 13	1	GRANADA

**Table 4:** Renal survival analysis in patients with *PKD1* variants.

Gene	c.DNA Designation	Protein Change	Described in DB	*n*	Mean Age RRT	SD	Median	%
*PKD1*	c.2534C>T	p.Leu845Ser	Described	14	70.2	63.30–77.14	72.8	61.16–84.46
*PKD1*	c.2591delG	(p.Gly864Alafs*34)	Not described	7	57.6	47.22–68.04	54.9	50.04–59.73
*PKD1*	c.3162-?_6915+?del	p.-	Not described	7	78.1	65.06–91.03	83.0	33.57–132.44
*PKD1*	c.4988_4990del	p.Ser1663del	Not described	10	62.2	60.43–63.91	62.6	59.23–65.95
*PKD1*	c.5013_5031del	(p.Asp1671Glufs*45)	Not described	10	69.6	60.77–78.47	65.3	61.63–68.89
*PKD1*	c.6005T>A	p.Val2002Asp	Not described	9	77.1	71.56–82.71	76.2	71.65–80.81
*PKD1*	c.6791C>A	p.Ser2264*	Described	9	51.8	45.34–58.40	53.9	44.94–63.02
*PKD1*	c.7292T>A	(p.Leu2431Gln)	Described by our group	57	66.4	63.04–69.73	67.0	60.20–73.91
*PKD1*	c.7480G>T	(p.Glu2494*)	Not described	6	48.1	28.38–67.68	55.8	34.51–77.24
*PKD1*	c.7784_7785dup	(p.Leu2596Cysfs*25)	Not described	8	52.6	44.84–60.41	54.6	38.28–70.87
*PKD1*	c.8579delA	p.Gln2860Argfs*1	Described	7	64.5	57.82–71.26	68.6	48.65–88.58
*PKD1*	c.8819C>T	p.Pro2940Leu	Described	34	63.6	59.54–67.69	65.3	61.51–69.04
*PKD1*	c.9202-16G>A	p.Pro3067fs*	Described	27	71.1	61.77–80.40	82.2	64.97–99.52
*PKD1*	c.9380G>T	(p.Gly3127Val)	Not described	6	62.5	61.15–63.81	61.9	61.09–62.75
*PKD1*	c.9616C>T	p.Gln3203*	Described	10	73.7	61.52–85.98	68.9	64.41–73.59
*PKD1*	c.10406-7C>T	p.-	Not described	8	71.6	56.90–86.42	70.6	24.89–116.28
*PKD1*	c.10441delG	(p.Val3481Serfs*46)	Not described	10	51.2	43.91–58.58	52.6	48.65–56.49
*PKD1*	c.10527_10528delGA	p.Glu3509Aspfs*117	Described	117	64.8	61.72–68.03	69.4	60.32–78.45
*PKD1*	c.10958C>T	p.Ala3653Val	Described	5	73.1	65.04–81.15	75.9	58.44–93.39
*PKD1*	c.11294_11313del20	(p.Pro3765Argfs*44)	Not described	28	56.3	48.65–64.04	59.3	47.99–70.70
*PKD1*	c.11456A>G	p.Tyr3819Cys	Described	12	66.9	62.38–71.42	69.8	68.53–71.06
*PKD1*	c.11512C>T	p.Gln3838*	Described	6	55.1	49.99–60.14	57.7	43.36–72.09
*PKD1*	c.12269C>T	p.Arg4020*	Described	8	61.1	59.54–62.49	62.0	59.45–64.54

**Table 5:** Renal survival analysis in patients with *PKD2* variants.

Gene	c.DNA Designation	Protein Change	Described in DB	n	Mean Age RRT	SD	Median	%
*PKD2*	c.1807dup	p.Met603Asnfs*23	Not described	14	72.87	66.09–79.66	71.1	68.85–73.36
*PKD2*	c.1445delT	p.Phe482Serfs*32	Described	20	73.60	63.43–83.77	82.0	51.84–112.15

**Table 6:** Comparison of renal survival curves in *PKD1*.

	Type of Variant on *PKD1* Gene	*n*	Median (Years)
Age RRT	Truncating (nonsense/frameshift)	136	51 ± 8.9
	Non-truncating (missense)	91	54.5 ± 10.7
Age of patient death	Truncating	105	54.7 ± 14.3
	Non-truncating	99	62.5 ± 10.67

## Data Availability

All data generated or analyzed during this study are included in this article. Further enquiries can be directed to the corresponding author.

## Supplementary Materials

The following supporting information can be downloaded at: https://www.mdpi.com/article/10.3390/jcm13061751/s1, Figure S1: Family 47: We have information for 4 generations, a total of 28 members related to the oldest case, born in Pórtugos. Six members suffer from the disease, four males and two females; the remaining members have not been genetically studied, including 4 descendants of diagnosed individuals. Three affected individuals have tested positive for the genetic variant c.7292T>A. Genetic testing has been conducted on one healthy member who does not carry the mentioned variant. Of the two deceased, one was in TRS. Figure S2: Family 48: Originally from Órgiva, we have information for 6 generations, totalling 71 members, of whom 31 suffer from PQRAD (20 females; 11 males). We have 11 relatives for whom we have neither clinical nor genetic data. Among the deceased relatives, 41.7% underwent TRS, and 100% had CKD. Genetic testing has been performed in this family on 7 affected members, all tested positive for the variant c.7292T>A, and on 5 unaffected members who do not carry it. Figure S3: Family 49: We have compiled data from 5 generations, totalling 24 members related to the considered ancestor, born in Pórtugos. Among them, the disease has been diagnosed in 6 individuals (4 females and 2 males), with genetic testing confirming the presence of the variant c.7292T>A in 2 of them, and one unaffected member not showing the variant in genetic testing; the others have not been studied. Of the affected individuals, 3 have passed away, and all of them attended TRS. Figure S4: Family 50: Coming from Pórtugos, it consists of 52 members, of which we know for certain that 19 suffer from PQRAD (6 males; 13 females); the remaining 6 members have not been studied. Of the deceased affected individuals, 50% underwent TRS. In this family, genetic studies have been conducted on 7 affected members, all of whom have the variant, and one study on a healthy relative who does not have it. Figure S5: Map with the geographical distribution of the variants analyzed by our group in the province of Granada.
