# Supplementary material for: Clinical Utility of Genetic Testing with Geographical Locations in ADPKD: Describing New Variants
Source: J Clin Med. 2024 Mar 18;13(6):1751. doi: 10.3390/jcm13061751 (PMC10971467; doi:10.3390/jcm13061751)
Supplement: Supplementary file 1 [file jcm-13-01751-s001.zip › jcm-2891906-supplementary.pdf]

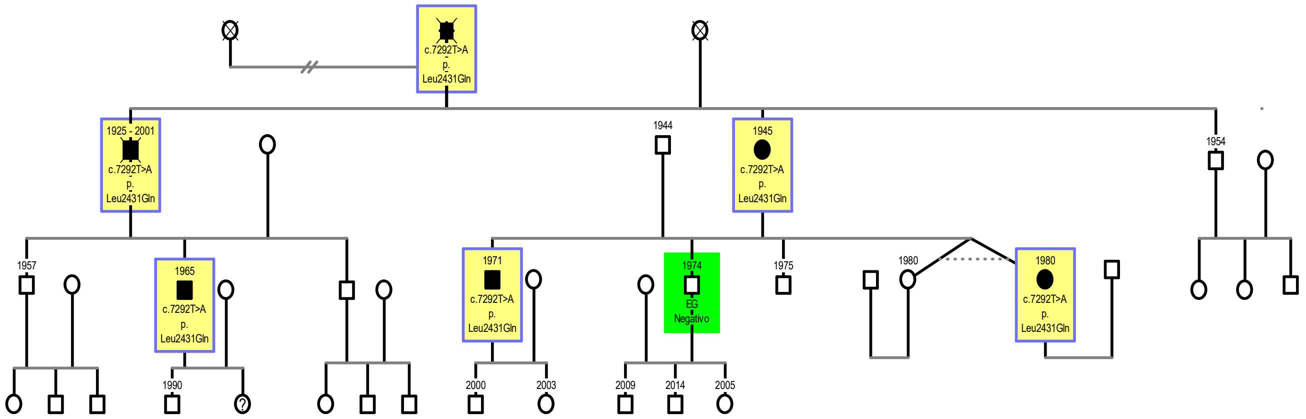

Figure S1. - Family 47: We have information for 4 generations, a total of 28 members related to the oldest case, born in Pórtugos. Six members suffer from the disease, four males and two females; the remaining members have not been genetically studied, including 4 descendants of diagnosed individuals. Three affected individuals have tested positive for the genetic variant c.7292T>A. Genetic testing has been conducted on one healthy member who does not carry the mentioned variant. Of the two deceased, one was in TRS.

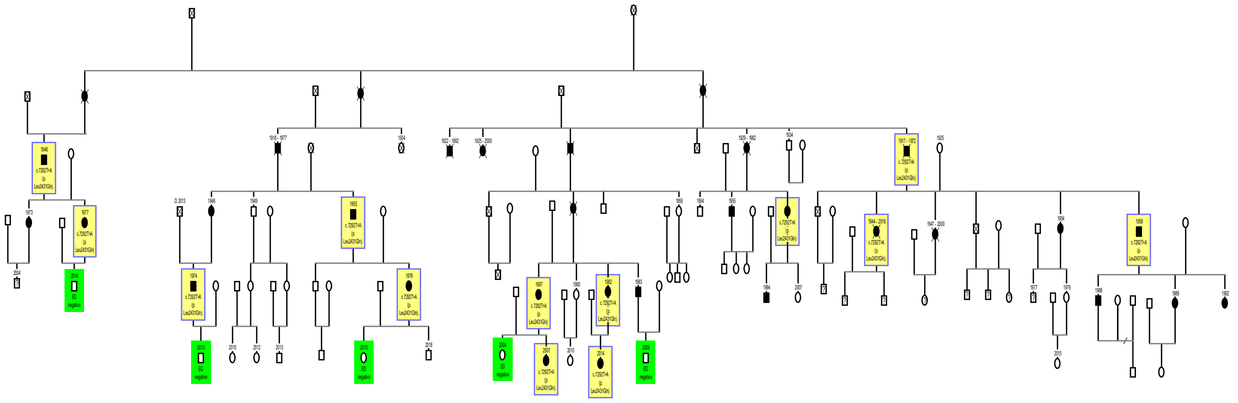

Figure S2. - Family 48: Originally from Órgiva, we have information for 6 generations, totalling 71 members, of whom 31 suffer from PQRAD (20 females; 11 males). We have 11 relatives for whom we have neither clinical nor genetic data. Among the deceased relatives, 41.7% underwent TRS, and 100% had CKD. Genetic testing has been performed in this family on 7 affected members, all tested positive for the variant c.7292T>A, and on 5 unaffected members who do not carry it.

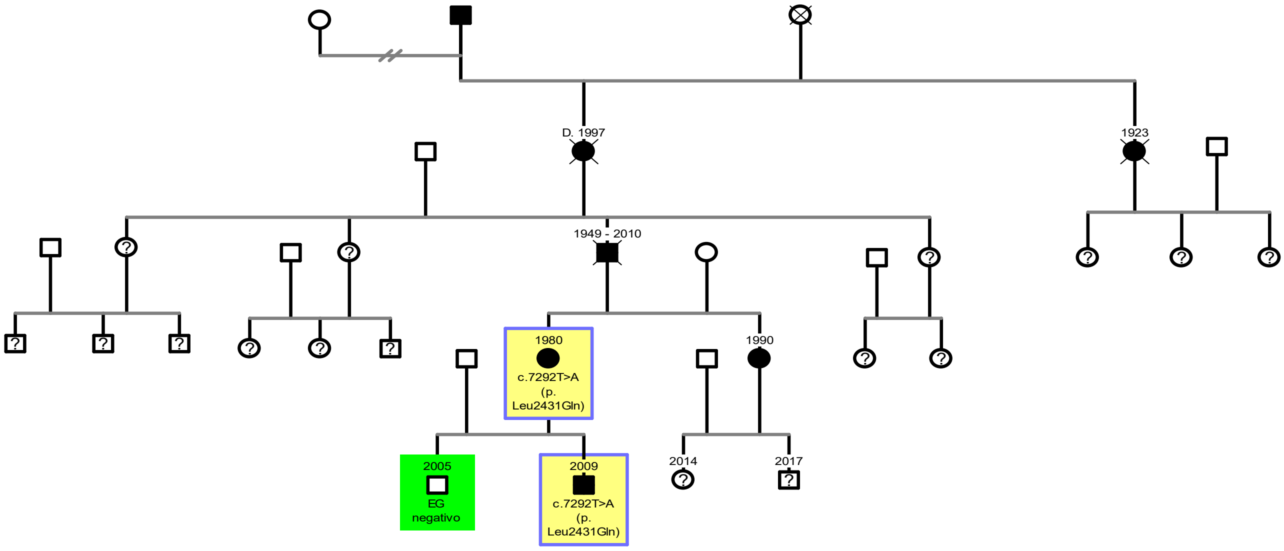

Figure S3. - Family 49: We have compiled data from 5 generations, totalling 24 members related to the considered ancestor, born in Pórtugos. Among them, the disease has been diagnosed in 6 individuals (4 females and 2 males), with genetic testing confirming the presence of the variant c.7292T>A in 2 of them, and one unaffected member not showing the variant in genetic testing; the others have not been studied. Of the affected individuals, 3 have passed away, and all of them attended TRS.

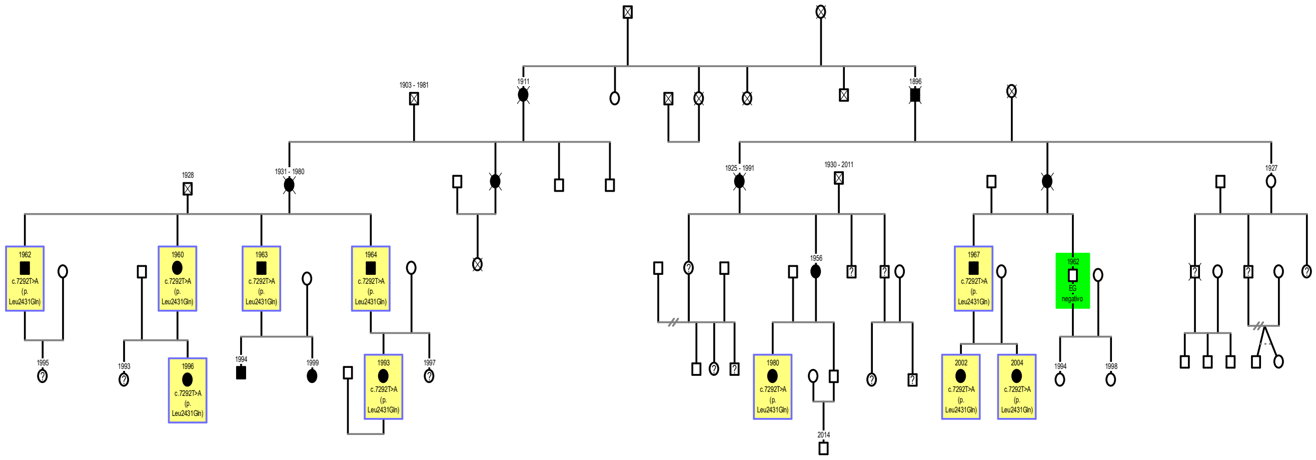

Figure S4. - Family 50: Coming from Pórtugos, it consists of 52 members, of which we know for certain that 19 suffer from PQRAD (6 males; 13 females); the remaining 6 members have not been studied. Of the deceased affected individuals, 50% underwent TRS. In this family, genetic studies have been conducted on 7 affected members, all of whom have the variant, and one study on a healthy relative who does not have it.

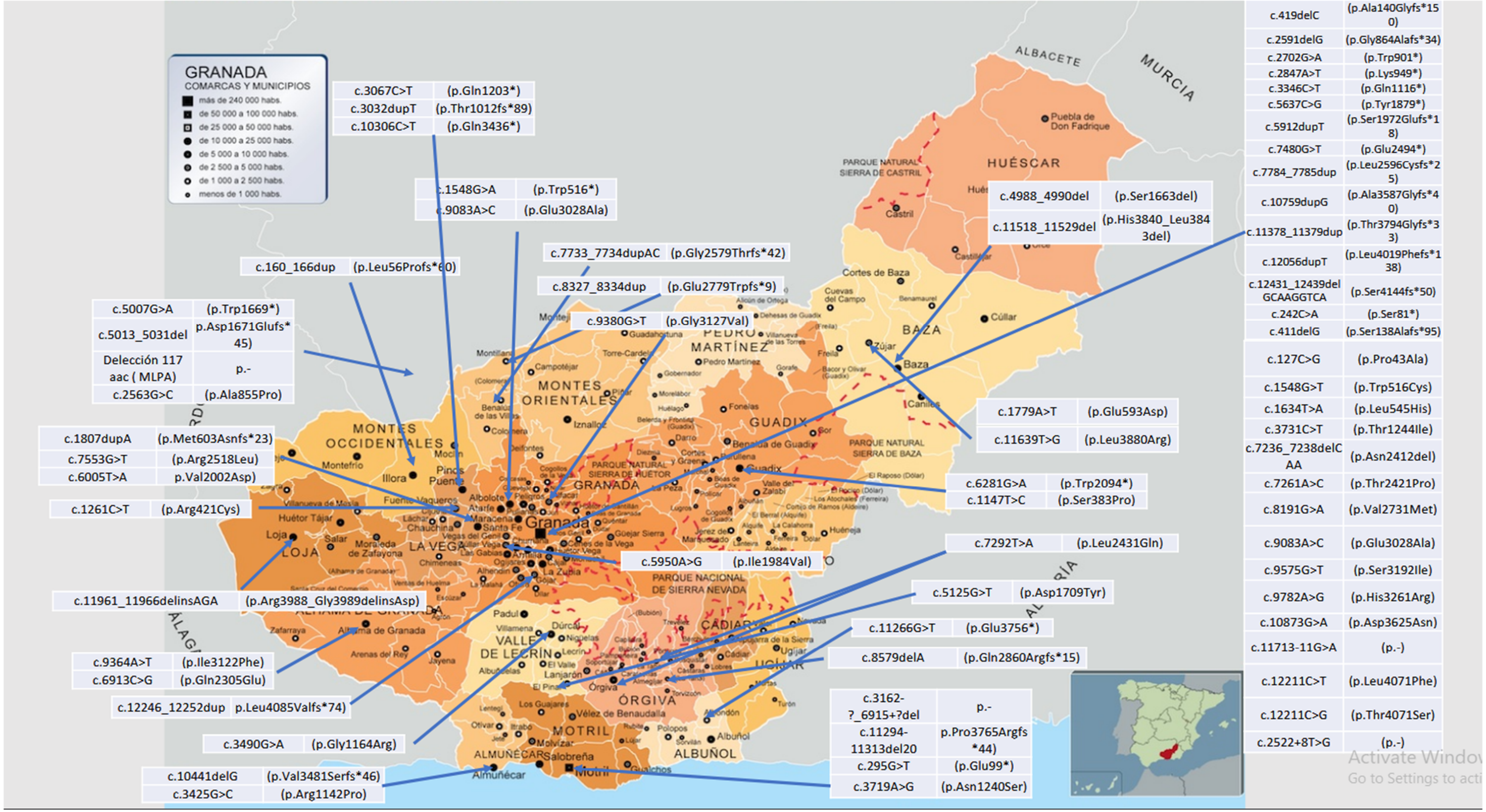

Figure S5. Map with the geographical distribution of the variants analyzed by our group in the province of Granada.
